# Predictors of university students’ leisure participation within the context of the theory of planned behavior

**DOI:** 10.3389/fpsyg.2026.1899265

**Published:** 2026-09-10

**Authors:** Gül Yağar, Faruk Yamaner, Tennur Yerlisu Lapa

**Affiliations:** 1 Coaching Education, Faculty of Sport Science, Hitit University, Çorum, Türkiye; 2 School of Physcial Education and Sport, Esenyurt University, Istanbul, Türkiye; 3 Recreation, Faculty of Sport Science, Akdeniz University, Antalya, Türkiye

**Keywords:** attitude, involvement, leisure, motivation, negotiation

## Abstract

**Background:**

University students’ participation in leisure activities during their transition to adulthood supports their psychological, social, and physical development, and the theory of planned behavior (TPB) offers a framework to explain this. However, the literature shows that the contribution of leisure attitudes, motivation, and negotiation strategies to students’ leisure participation and exercise participation has not been adequately addressed.

**Methods:**

This cross-sectional study, within the framework of the theory of planned behavior, examined the effects of leisure motivation, attitudes, negotiation strategies, and participation levels on the leisure behaviors of undergraduates (*n* = 709) enrolled in sports science faculties using linear and hierarchical regression analyses.

**Results:**

Leisure attitudes, motivation, and negotiation strategies significantly predicted participation levels (*p* < 0.001) and together explained 33% of the variance. Hierarchical regression analysis indicated that participation, negotiation strategies, attitudes, and motivation significantly predicted engagement and that participation, negotiation strategies, attitudes, and motivation significantly predicted participation in leisure exercise (*p* < 0.001).

**Conclusion:**

Leisure attitudes, motivation, and negotiation strategies indicate that university students’ participation in leisure activities influences their engagement in those activities, and this participation can help explain their level of leisure exercise. These results suggest that the theory of planned behavior can offer a useful framework for understanding leisure behaviors and that supporting motivation and negotiation strategies can contribute to increasing students’ participation in leisure activities.

## 1 Introduction

Individuals’ participation in leisure activities plays a critical role in their daily living skills (Chang et al., 2018; Chein, 2017; Eskiler et al., 2019; Kim et al., 2018). For individuals transitioning from adolescence to adulthood and attending university, participation in leisure activities during this transition phase holds significant importance for the development of their psychological, social, physical, and sense of belonging (Morris and Orton-Johnson, 2024; Takiguchi et al., 2023). When considering the theoretical foundations addressing participation in leisure activities, the theory of planned behavior, widely used in the literature, is among the most common approaches (Ajzen and Driver, 1992; Ramlan et al., 2023; Arnold et al., 2024). The theory of planned behavior examines the factors influencing behavior. These factors consist of the underlying intention and the components influencing it: attitude, subjective norms, and perceived behavioral control. When these components are examined, it is found that attitude—an indicator of how an individual makes sense of their experiences, the value they attribute to an activity (Funk et al., 2008), and their knowledge, beliefs, and feelings (Freire and Fonte, 2007)—influences behavior and supports the understanding of it (Hsieh et al., 2004). Subjective norms are related to an individual’s social perceptions (Fishbein and Ajzen, 2011) and determine their motivation by influencing their positive and negative perspectives in the decision-making process (Bless et al., 1992). Perceived behavioral control, on the other hand, expresses an individual’s belief in their ability to exert effort, maintain it, and show resilience in the face of difficulties (Bandura, 1977). Together with these components, a comprehensive theoretical framework is formed to explain behavior.

When the theory of planned behavior is considered in the context of leisure time, studies in the literature highlight that among the most frequently discussed issues regarding individuals’ participation in leisure activities are attitudes (Akçakese et al., 2025; Niu et al., 2025; Han et al., 2025), negotiation strategies (Chen et al., 2025; Li et al., 2025; Chun et al., 2025), motivation (Liang et al., 2025; Luong and Ho, 2025; Lim and Kim, 2026), and involvement (Budak and Yumuk, 2025; Tükel et al., 2025; Zhou et al., 2025). Attitude also has a significant influence in shaping leisure behaviors (Güçlü, 2013; Nelson et al., 2010) and guiding preferences (Freire and Fonte, 2007). In this study, the attitude dimension, which is one of the factors influencing intention according to the theory, was assessed using the leisure attitudes scale. Subjective norm, another component of the theory, deals with a perceptual and motivational process that determines an individual’s approach to a behavior based on expectations from others and social pressures (Fishbein and Ajzen, 2011). In motivational processes, the theory supports the influence of perception on both external sources (Roberts et al., 2007) and internal sources within the individual (Dann, 2016). Motivation is one of the constructs most closely related to subjective norms in TPB, and this concept has been addressed from various theoretical approaches. Self-determination theory distinguishes intrinsic motivation, various forms of extrinsic motivation, and amotivation, positioning them along a continuum of self-determination regulation (Deci and Ryan, 1985; Vallerand, 1997). Achievement goal perspectives relate motivation to the goals individuals adopt when engaging in an activity, such as mastery and performance orientation (Nicholls, 1984), while needs-based approaches emphasize autonomy, competence, and relationship satisfaction as driving forces for sustained participation (Deci and Ryan, 2000). Subjective norm, in its original formulation, briefly refers to the perceived social pressure to perform or not perform a behavior (Fishbein and Ajzen, 2011); the Leisure Motivation Scale (LMS) used here does not directly measure this perceived pressure but instead measures intrinsic, extrinsic, and amotivational orientations, which in this study are assumed to arise in part as a response to such normative influences (Deci and Ryan, 2000; Pelletier et al., 1996). Evaluating this process through the lens of leisure motivation supports explaining this component of leisure behavior. In terms of leisure time, participation supported by intrinsic motivation leads to deeper and more sustainable engagement, while extrinsically regulated or unmotivated participation tends to predict weaker or shorter-lived engagement (Pelletier et al., 1996). Applied to university life, this multifaceted view of motivation suggests that fostering autonomous and defined forms of motivation, rather than relying solely on external pressures, may be a more effective strategy for promoting sustainable leisure and physical activity participation among students. Therefore, the scales used in this study are not direct measurements of classical TPB constructs, but conceptually related to them.

Perceived behavioral control, the final component of intention that can directly affect behavior, refers to the constraints an individual may encounter in the process and their ability to overcome them (Chatzisarantis and Hagger, 2005). Therefore, it is conceptually justifiable to associate it with negotiation skills within the concept of leisure time. These negotiation skills are assessed in terms of time, financial, social, and structural constraints. This assessment demonstrates an individual’s ability to cope with both internal and external constraints (Crawford et al., 1991). The Leisure Negotiation Strategies Scale (LNSS) is considered a measurable representation of this construct because negotiation strategies reflect the cognitive and behavioral efforts individuals make to overcome perceived leisure constraints; this scale is conceptually consistent with self-efficacy in the face of obstacles, even though the two constructs are not identical (Crawford et al., 1991; Hubbard and Mannell, 2001). The LNSS is based on working assumptions rather than exact equivalences, and this gap is noted in the Limitations section.

**Figure 1:**
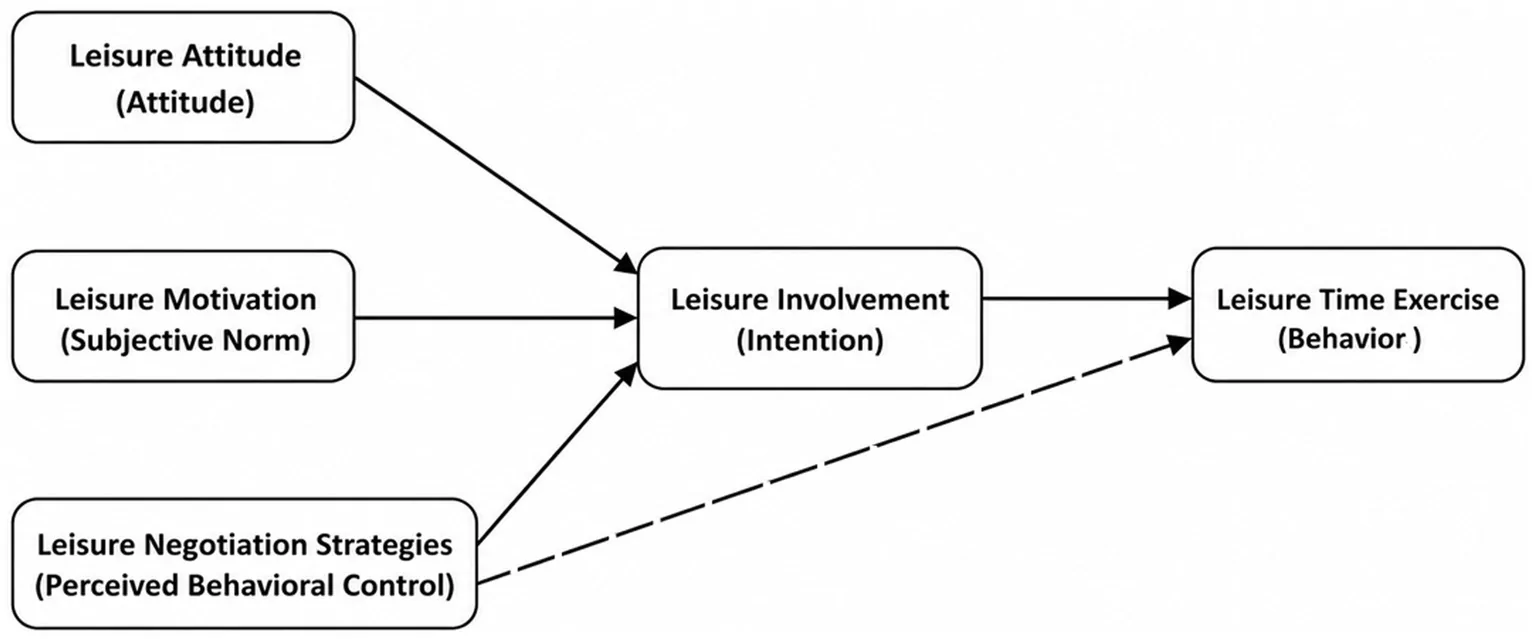
Conceptual model based on the theory of planned behavior (Ajzen, 1991).

In the theory of planned behavior, intention is a significant determinant in explaining leisure behavior (Ajzen, 1985). When evaluated in light of the leisure literature, this study shows that it is an important psychological state reflecting an individual’s participation, interest in activities, commitment, and level of meaning-making (Kyle and Chick, 2004; Kim et al., 2009). In evaluating participation, the degree of personal meaning and importance it holds for the individual (Ko et al., 2009) can be considered the closest evaluator of intention among the determinants of behavior. Individuals’ preference for physical activities over passive activities in their leisure time helps prevent diseases and maintain a healthy body, while also increasing the pleasure, satisfaction, and positive experiences obtained (Kuykendall et al., 2015). Therefore, this study aims to evaluate the extent to which leisure time behavior is explained for university students entering young adulthood and to examine its effects on participation levels (Figure 1). Based on the theory of planned behavior, this study was created to check these research goals:

*H1*: LAS, LMS, and LNSS have an effect on the explanation of LIS.

*H2*: The subdimensions of LAS, LMS, and LNSS have an effect on the explanation of LIS.

*H3*: LIS, LAS, LMS, and LNSS have an effect on the explanation of the LTEQ.

*H4*: The subdimensions of LIS, LAS, LMS, and LNSS have an effect on the explanation of the LTEQ.

## 2 Methods

### 2.1 Research group

This cross-sectional study included students from the sports science faculties of five different universities as a sample. Students were deemed eligible for inclusion if they were officially undergraduates in the sports science faculty of one of the five participating universities and voluntarily agreed to participate after being informed about the study; however, students who did not complete the survey questions or whose data were identified as invalid or multivariate outliers during data screening were excluded from the study. The sample size was determined using the formula *n* = (N.p.q.Z^2^)/[(*N* − 1).d^2^ + p.q.Z^2^], encompassing a total of 4,032 students. With a 99% confidence interval and a 5% margin of error, the minimum sample size was calculated as 572. The sample size was determined using a stratified sampling method at the university level; in this method, the number of participants from each university (Table 1) was proportional to that university’s share of the total student population, and a total of 822 students were obtained, taking into account possible losses. After data collection, cases with incomplete or inconsistent responses were excluded, and the remaining data were examined for multivariate outliers using Mahalanobis distance; cases exceeding the critical chi-square value at *p* < 0.001 for the number of estimators were excluded from further analysis. Of the 822 volunteers who participated, 113 cases were excluded through this combined screening process (incomplete/inconsistent responses (*n* = 29) and multivariate outliers (*n* = 84)), and a total of 709 individuals participated in the study (female mean = 21.21 ± 2.45; male mean = 21.18 ± 2.12).

**Table 1:** Sample size calculation and participant recruitment.

University	Akdeniz Uni.	Amasya Uni.	Hitit Uni.	Ondokuz Mayıs Uni.	Tokat Gaziosmanpaşa Uni.	Total
Target population, *n* (%)	700 (17.4)	170 (4.2)	1,262 (31.3)	1,200 (29.8)	700 (17.4)	4,032 (100)
Min. required sample size (*n*)	97	29	177	172	97	572
Students invited (*n*)	150	100	250	250	150	860
Part. who consented (*n*)	124	95	238	240	125	822
Part. included in the final analysis (*n*)	111	55	216	221	106	709
Response rate (%)	82.7	95.0	95.2	96.0	83.3	95.6

The minimum required sample size for each university was calculated using proportional stratified sampling based on the size of the target population. Participants with incomplete questionnaires or identified as multivariate outliers were excluded from the final analyses.

### 2.2 Data collection

Data collection was conducted face-to-face after obtaining approval from the Hitit University Non-Interventional Ethics Committee in accordance with the Helsinki Declaration (Ethics Code: 2018/216). All participants were informed about the research objectives and data collection procedures, and informed consent was obtained prior to participation; participants were also informed that their participation was voluntary and that they could withdraw from the study at any time. Accordingly, those who voluntarily participated in the study were included in the data. During this time, no participant requested withdrawal. The following validated and frequently studied scales from the literature were used as data collection tools in this study.

#### 2.2.1 Leisure Time Exercise Questionnaire

The “Leisure Time Exercise Questionnaire (LTEQ)” (Godin and Shephard, 1985) was translated into Turkish by Yerlisu Lapa and Yağar (2015). In this study, the LTQ scale was used to determine the behavioral levels of students within the framework of Theoretical Planned Behavior (TPB). The questionnaire measures leisure time exercise by calculating the scores of individuals who participate in exercise during their leisure time. Those scoring 24 and above are considered active, those scoring 14–23 are considered moderately active, and those scoring 13 and below are considered not sufficiently active.

#### 2.2.2 Leisure Attitude Scale

The “Leisure Attitude Scale (LAS),” developed by Ragheb and Beard (1982) and confirmed to be valid and reliable by Akgül and Gürbüz (2011), was used to determine the attitude levels of students within the TPB. The scale is a 5-point Likert type consisting of 36 items in total and 3 sub-dimensions: (1) cognitive, (2) affective, and (3) behavioral. The minimum score calculated on the scale is 36; the maximum score is 180. In the reliability analysis conducted within the scope of this study, Cronbach’s alpha values were obtained as follows: cognitive sub-dimension: 0.911; affective sub-dimension: 0.908; behavioral sub-dimension: 0.881; and overall scale: 0.951.

#### 2.2.3 Leisure Involvement Scale

The Leisure Involvement Scale (LIS), originally developed by Kyle et al. (2007), was translated into Turkish by Gürbüz et al. (2018). LIS measures attraction, centrality, social bonding, identity affirmation, and identity expression using a 5-point Likert format. In the reliability analysis conducted within the scope of this study, Cronbach’s alpha values were obtained as follows: attraction sub-dimension: 0.762; social bonding sub-dimension: 0.620; identity affirmation sub-dimension: 0.591; identity expression sub-dimension: 0.674; centrality sub-dimension: 0.731; and overall scale: 0.897.

#### 2.2.4 Leisure Motivation Scale

To find out what motivated people to take part in leisure activities, Pelletier et al. (1996) created the Leisure Motivation Scale (LMS). This study utilized this scale to assess the students’ subjective norms within the TPB framework. Mutlu (2008) adapted the scale to Turkish. The scale adapted to Turkish consists of a total of 22 items and 5 sub-dimensions (knowing and achieving, amotivation, experiencing stimuli, external regulation, and identification/introjection). The statements in the scale were evaluated on a 5-point Likert-type scale ranging from “I completely disagree (1)” to “I completely agree (5).” In the reliability analysis conducted within the scope of this study, Cronbach’s alpha values were obtained as follows: amotivation sub-dimension: 0.796; knowing and achieving sub-dimension: 0.770; experiencing stimulation sub-dimension: 0.632; identification/introjection sub-dimension: 0.733; external regulation sub-dimension: 0.657; and overall scale: 0.864.

#### 2.2.5 Leisure Negotiation Strategies Scale

In the study, the students perceived behavioral control was assessed using the Leisure Negotiation Strategies Scale, on the grounds that negotiation strategies reflect an individual’s perceived capacity to overcome leisure constraints, consistent with the self-efficacy component of perceived behavioral control (Bandura, 1977). This scale, called the “Leisure Negotiation Strategies Scale (LNSS),” was created by Hubbard and Mannell (2001). Yerlisu-Lapa (2014) translated it into Turkish, and the scale has a 5-point Likert structure. In the reliability analysis conducted within the scope of this study, Cronbach’s alpha values were obtained as follows: time management sub-dimension: 0.728; skill acquisition sub-dimension: 0.837; interpersonal relationships sub-dimension: 0.773; internal approval sub-dimension: 0.552; physical fitness sub-dimension: 0.720; financial management sub-dimension: 0.702; and overall scale: 0.888.

### 2.3 Statistical analysis

The normality of the data was evaluated using both the Kolmogorov–Smirnov test and the skewness and kurtosis values (Table 2). Although the Kolmogorov–Smirnov test statistics and corresponding *p*-values were calculated for each scale, these significance-based normality tests are known to be highly sensitive to even minor deviations from normality in large samples such as the present study (*n* = 709) (Sayili and Gunver, 2025). Therefore, the assumption of normality was primarily assessed based on the skewness and kurtosis values, which fell within the acceptable range of ±1.5, rather than the Kolmogorov–Smirnov *p*-values (Tabachnick and Fidell, 2015). Subsequently, the TPB-based model was tested using linear and hierarchical regression analyses after confirming that the assumptions of regression were satisfied, including the absence of autocorrelation (Durbin–Watson < 2.0) and multicollinearity (1 < VIF < 4).

**Table 2:** Descriptive statistics of the datasets.

Variables	K-S statistic	Sd	p	Skewness	Kurtosis	Mean	Median
LIS	0.066	709	0.000	−0.533	0.275	3.85	3.93
LAS	0.056	709	0.000	−0.684	0.622	144.73	145.00
LMS	0.039	709	0.000	−0.170	0.791	3.50	3.47
LNSS	0.026	709	0.000	−0.159	0.506	3.64	3.63

## 3 Results

In the study conducted within the framework of the TPB, intention, the determinant of behavior, was analyzed from the outset. In this study, the effects of attitudes, subjective norms and perceived behavioral control, which constitute the first step of the model, on intention were analyzed through linear regression analysis (Table 2). In this context, the effects of the levels of leisure attitudes, motivation and negotiation strategies on the level of involvement were evaluated. As a result of the analysis, it was determined that the scales of leisure attitudes, motivations and negotiation strategies defined 33% of the level of involvement and were statistically significant (*R* = 0.575, *R*^2^ = 0.331). An analysis of the beta coefficients for the level of leisure revealed that the variable with the highest predictive value was leisure attitude (*β*=0.429; *p* < 0.001), and the lowest predictor was leisure negotiation (*β*=0.031; *p* < 0.001). When the coefficients of the independent variables are considered, the significant predictive power on participation in leisure activities indicates that hypothesis H1 is accepted (Table 3).

**Table 3:** Regression analysis results of the effects of scale on LIS.

Variables	*R*	*R* ^2^	Adj. *R*^2^	*B*	*Std. Error*	*𝛽*	*F*
	0.575	0.331	0.328				116.231*
LAS				0.014	0.001	0.429	
LMS				0.238	0.049	0.209	
LNSS				0.038	0.051	0.031	

*p < 0.001; 1 < VIF<5; Durbin Watson = 1.492.

When evaluated in terms of subdimensions, the variables (*R* = 0.603, *R*^2^ = 0.364) statistically significantly defined 36% of the students’ level of leisure involvement on the basis of the model (*R*^2^ = 0.331). When the beta (𝛽) coefficients were reviewed to determine the definitive power of the subdimensions of the scales in the model, the cognitive dimension (*β* = 0.216, *p* < 0.001) and the affective dimension (*β* = 0.195; *p* < 0.001) variables, which are the subdimensions of the leisure attitude scale, had the highest definitive power (Table 4). Based on the results obtained, hypothesis H2 has been accepted. When testing the beta coefficients in Table 3, it was found that there was a very small negative impact on the aspects of behavior, identity/introspection, time management, and financial management.

**Table 4:** Regression analysis results of the effects of the scale subdimensions on LIS.

Variables	*R*	*R* ^2^	Adj. *R*^2^	*B*	Std. Error	*β*	*F*
	0.603	0.364	0.351				28.370*
Cognitive				0.018	0.005	0.216	
Affective				0.017	0.005	0.195	
Behavioral				−0.001	0.004	−0.012	
Amotivation				0.075	0.025	0.131	
Knowing and achieving				0.128	0.051	0.137	
Experiencing stimulus				0.090	0.044	0.101	
Identification/Introjection				−0.013	0.059	−0.013	
External regulation				0.023	0.032	0.030	
Time management				−0.057	0.032	−0.065	
Gaining skill				0.063	0.038	0.072	
Interpersonal relationships				0.022	0.032	0.027	
Internal approval				0.030	0.032	0.036	
Physical fitness				0.036	0.031	0.044	
Financial management				−0.041	0.036	−0.047	

*p < 0.05; 1 < VIF<5; Durbin Watson = 1.499.

In the first step of the hierarchical regression analysis conducted to determine students’ leisure time exercise participation (LTEQ) within the context of the Theory of Planned Behavior (TPB), leisure involvement level (LIS) was included in the model, and it was determined that this variable has significant explanatory power on exercise participation (*R* = 0.221; *R*^2^ = 0.049, *p* < 0.001). In the second step of the analysis, leisure negotiation strategies (LNSS) were added to the model, but the inclusion of this variable did not lead to a change in the variance explained, and its direct effect on exercise frequency was found to be insignificant (*R* = 0.221; *R*^2^ = 0.049, *ε* = −0.014, *p* > 0.05). In the third and final step of the hierarchical model, leisure time attitudes (LAS) and leisure time motivation levels (LMS) were integrated into the analysis, completing the process (*R* = 0.231; *R*^2^ = 0.053). When the coefficients of the independent variables are considered, the significant predictive power indicates that hypothesis H3 is accepted (Table 5).

**Table 5:** The results of the hierarchical regression analysis of the scales on the LTEQ.

Step	Variables	*R*	*R* ^2^	Adj. *R*^2^	*B*	Std. Error	*β*	*F*
1		0.221	0.049	0.047				36.227*
	LIS				6.261	1.04	0.221	
2		0.221	0.049	0.046				18.155*
	LIS				6.402	1.114	0.226	
	LNSS				−0.477	1.340	−0.014	
3		0.231	0.053	0.048				9.883*
	LIS				7.132	1.271	0.252	
	LAS				−0.070	0.041	−0.078	
	LMS				1.313	1.675	0.041	
	LNSS				−0.016	1.713	−0.016	

*p < 0.05; 1 < VIF<5; Durbin Watson = 1.856.

Examining the regression coefficients and model summaries in Table 5, it is seen that leisure involvement (LIS), which was included in the model in the first stage of the hierarchical regression analysis, is the variable that provides the highest explanatory power on the dependent variable with approximately 5%. Considering the standardized beta coefficients, the leisure involvement dimension is the most important predictor positively affecting leisure time exercise participation, and this positive effect gradually increased with the addition of subsequent variables to the model (*β*1 = 0.221 < *β*2 = 0.226 < *β*3 = 0.252, *p* < 0.001). While leisure time attitude (LAS), which was included in the model in the last step, had a low-level negative and significant effect on exercise participation (*β* = − 0.078, *p* < 0.05), the effect of leisure time motivation (LMS) was found to be statistically insignificant. Following these findings obtained from the overall scale scores, the analyses were repeated at the sub-dimension level in order to examine the relationships more deeply (Table 6). Based on the coefficients of the independent variables, their significant predictive power indicates that hypothesis H4 is accepted (Table 6).

**Table 6:** Hierarchical regression analysis results of the scale subdimensions on LTEQ.

Step	Variables	*R*	*R* ^2^	Adj. *R*^2^	*B*	Std. Error	*𝛽*	*F*
1		0.250	0.063	0.057				11.762*
	Attraction				0.792	1.329	0.032	
	Centrality				4.211	1.056	0.195	
	Social bonding				−0.852	1.156	−0.036	
	Identity affirmation				0.792	1.329	0.032	
	Identity expression				1.928	1.231	0.080	
2		0.276	0.076	0.063				5.741*
	Attraction				1.043	1.363	0.042	
	Centrality				4.143	1.063	0.192	
	Social bonding				−0.874	1.181	−0.037	
	Identity affirmation				1.043	1.363	0.042	
	Identity expression				1.943	1.237	0.081	
	Time Management				−1.373	1.032	−0.055	
	Skill-acquisition				−1.617	1.133	−0.065	
	Inter-personal relations				1.453	1.081	0.062	
	Intra-personal validation				−1.812	1.063	−0.077	
	Physical fitness				2.018	1.047	0.086	
	Financial management				0.615	1.212	0.025	
3		0.299	0.090	0.066				3.772*
	Attraction				1.726	1.395	0.070	
	Centrality				4.000	1.071	0.185	
	Social bonding				−0.653	1.191	−0.028	
	Identity affirmation				1.726	1.395	0.070	
	Identity expression				1.792	1.284	0.075	
	Cognitive				−0.302	0.161	−0.125	
	Affective				0.060	0.172	0.025	
	Behavioral				0.044	0.123	0.019	
	Amotivation				−1.161	0.861	−0.071	
	Knowing and accomplish				1.142	1.764	0.043	
	Experiencing stimulation				0.671	1.507	0.027	
	Identified/Introjected				−1.721	2.025	−0.061	
	External regulation				2.416	1.124	0.112	
	Time management				−1.804	1.095	−0.072	
	Skill-acquisition				−1.129	1.314	−0.045	
	Inter-personal relations				1.202	1.104	0.051	
	Intra-personal validation				−1.765	1.101	−0.075	
	Physical fitness				1.936	1.050	0.083	
	Financial management				0.624	1.239	0.025	

*p < 0.05; 1 < VIF<10; Durbin Watson = 1.887.

The subdimension of the leisure involvement scale that was included in the model in the first step was 6% definitive (*R* = 0.250; *R*^2^ = 0.063), the subdimension of the leisure negotiation strategies scale, which was included in the modeling the second step, was 8% definitive (*R* = 0.276; *R*^2^ = 0.076), and the subdimension of the Leisure Attitude and Motivation Scale, which was included in the third step, was 9% definitive (*R* = 0.299; *R*^2^ = 0.090). When the sub-dimensions are considered, although each sub-dimension is generally significant, the centrality sub-dimension, which is a sub-dimension of leisure involvement, has formed a statistically significant unit at every step. This suggests that, from the perspective of the participating individuals, the activities they engage in constitute an important part of their lives (Table 6).

## 4 Discussion

Lewin (1951: cited by Hausenblas et al., 1997) stated that “there is nothing more practical than a good theory,” and the theory of planned behavior is a good one (Hausenblas et al., 1997). Based on this, TPB offers a functional perspective in explaining the leisure time use patterns of university students. Accordingly, examining how university students participate in leisure time within the context of TPB clearly reveals that psychological constructs and behavioral intentions are interconnected through complex networks. Individuals’ attitudes toward leisure activities, their motivations, and negotiation strategies play a role in shaping their participation levels and explain a portion of the variance in future behavioral planning. The fundamental proposition of TPB is that attitudes toward behavior, subjective norms, and perceived behavioral control directly shape behavioral intention. The literature shows that attitudes and self-efficacy perceptions have a strong influence on intentions toward leisure sports; This confirms that the intensity of motivational elements and leisure activities are also fundamental dynamics guiding behavioral intentions. In the evaluation of these factors within the scope of the hierarchical regression model, it was determined that the modules containing the control variables, which were added first to the analysis, had the lowest effect on the dependent variable. This situation can be explained by the fact that the ways in which university students manage their time and financial resources during their adjustment and adaptation processes to a new life stage show a dynamic structure that depends on environmental and situational changes (Sönmez and Gürbüz, 2022).

The findings from the current study demonstrate the interaction and explanatory power of the components of behavioral intention (BI) that affect leisure time participation among university students. The analysis results initially revealed a direct impact of the components of behavioral intention, considered at the main scale level, on the level of leisure time participation (Hypothesis H1). The findings showed that leisure time attitude, motivation, and negotiation strategies collectively explained 33% of the variance in leisure time participation (*R*^2^ = 0.33); this rate corresponds to approximately one-third of the participation level depending on the intention of the sample group. When the analysis was extended to include all sub-dimensions of the variables, the explanatory power was found to increase to 36% (*R*^2^ = 0.36) (Hypothesis H2). Although a holistic study testing these three basic factors (attitude, motivation, and negotiation strategies) simultaneously and together was not found in the literature, the current findings show a high degree of agreement with the literature when examining the individual relationships among the variables. Indeed, leisure attitudes (Kokkinaki and Lunt, 1997), leisure motivation (Alexandris, 2013; Lu and Schuett, 2014), and negotiation strategies (Wood and Danylchuk, 2015; Ma et al., 2024) are known to have independent, positive, and significant effects on participation levels. Similarly, strong leisure attitudes and high self-efficacy perceptions have been reported to directly trigger intentions toward leisure sports, thereby increasing actual participation levels (Lee and Park, 2021). The fact that perceived behavioral control significantly predicts individuals’ participation in leisure activities also empirically supports the theoretical model of TPB (Kim and Kang, 2021). Furthermore, leisure motivation is known to play a decisive role in both the general well-being and leisure participation frequency of university students (Xue et al., 2022). This positive impact of motivational processes on intention and behavior parallels the work of Di Battista et al. (2018), who discussed how motivational climates strongly influence students’ intentions to participate in physical activities. At this point, the importance of strategies for overcoming (negotiating) constraints in leisure time participation is undeniable; effective negotiation mechanisms increase both participation and satisfaction derived from the process. Furthermore, the literature shows that high levels of self-efficacy and positive attitudes toward retirement are directly influenced by individuals’ ability to effectively use negotiation strategies in leisure time participation (Lee et al., 2020).

In the hierarchical regression analysis based on TPB conducted within the scope of the research, no statistically significant increase in the total variance explained was observed in the second step (Step 2) of the model established through the main scale scores (Hypothesis H3). However, when the analysis was deepened by including the sub-dimensions of the scales (Hypothesis H4), a statistically significant, but low-level increase in the effect (*ΔR^2^*) on students’ exercise participation was detected between the hierarchical steps. This finding is consistent with studies in the literature indicating that TPB is an extremely flexible model in explaining different behavioral situations and contexts but that the explanatory power of the model can become much more effective by specifically including the sub-dimensions in the model (Abdullah et al., 2022). Nevertheless, considering individual and cultural differences is a critical requirement when applying the TPB framework. It is known that cultural orientations such as individualism and collectivism significantly influence the functioning of TPB models, the weights of variables, and the meanings individuals attribute to culturally existing behaviors (Feng et al., 2023). A broad literature review reveals that the variance ratios explained by TPB-based regression models vary widely in empirical studies. For example, in the pioneering study by Ajzen and Driver (1992), the explained variance of exercise participation intentions among university students was found to be approximately 78%. In a study conducted by Kerner and Kurrant (2003) on 129 female high school students, it was determined that attitude, motivation, and perceived behavior explained 50% of the intention variance, while intention and perceived behavioral control predicted actual behavior at the 5% level. Latimer and Martin Ginis (2005), working with individuals with spinal cord injuries, showed that attitude, motivation, and perceived behavioral control explained 60% of the intention variance. Another study of 80 patients with chronic kidney failure reported that intention and perceived behavioral control explained 27% of the variation in exercise behavior (Eng and Martin Ginis, 2007). A study on children found that attitude, subjective norm, and perceived behavioral control explained 33% of the variance in intention; intention was the most significant predictor of actual exercise behavior at 9%, followed by perceived behavioral control, attitude, and subjective norm, respectively (Wang and Wang, 2015). Given this diversity in the literature, the present study clearly shows that leisure time participation among university students is statistically significantly predicted by attitude, motivation, and negotiation strategies, explaining approximately one-third of the intention to participate. However, when evaluated in terms of overall leisure time exercise, this effect size is not very high. This finding is thought to be due to the fact that the sample group participating in the study consisted of a group from different universities and geographical regions, exhibiting high heterogeneity in terms of leisure time habits and opportunities, rather than a single homogeneous population.

### 4.1 Limitations and future prospects

This study is limited by several constraints. First, it is limited by the scales addressed within the framework of theory. Second, all data collection tools used in the research are limited to self-report questionnaires. Third, although the sample group was drawn from five universities, participants were limited to sports science faculties; this may limit the generalizability of the findings to university students in other fields of study. Fourth, the heterogeneity of the sample across different regions and universities should be considered, as it may have affected some of the observed values. Another conceptual limitation concerns the association of TPB constructs: Leisure attitude is used in relation to the attitude component included in theory; the Leisure Motivation Scale is used in relation to the motivational consequences of subjective norms rather than a direct measure of perceived social pressure, the Leisure Negotiation Strategies Scale is used in relation to perceived behavioral control rather than a validated TPB measure, and Leisure Involvement Scale is used in relation to the intention component included in theory. This reveals the final limitation of the study.

Future studies could investigate whether TPB differs within institutional or regional contexts. The theory can be considered within the context of a single region, institution, or field of study. Perceived constraints and environmental factors not included in the model, and their effects on behavior, can be examined. In this study, considering the survey fatigue that participants may have experienced, this weakness can be prevented by adjusting the number of questions.

## 5 Conclusion

Physical activity, a desirable state for leisure time participation, is particularly important for the healthy development of young people attending university. In this context, a hierarchical regression model, applied within the framework of the Theory of Planned Behavior (TPB), aimed to determine the linear relationships between leisure time attitudes, motivations, negotiation abilities, interests, and exercise participation among university students receiving sports training. The research results indicated that positive attitudes, strong motivational sources, and the ability to effectively cope with encountered problems, when present together in a positive interaction, increase and sustain leisure-time exercise participation. Theoretically, it was determined that attitude, subjective norm, and perceived behavioral control components have a positive role in students’ exercise intentions; and specifically in the behavioral dimension, the combined effect of intention and perceived behavioral control produced similar results to the overall effect level when attitude and subjective norm were also included in the model. However, it was found that the explanatory power of both intention and behavior reached higher levels when considered at the sub-dimension level than when compared to the overall scores of the scales. In conclusion, increasing students’ leisure involvement and ensuring their continued participation in exercise depend on the multifaceted and specific support of these psychosocial sub-dimensions.

## Data Availability

The original contributions presented in the study are included in the article/Supplementary material, further inquiries can be directed to the corresponding author.

## References

[ref1] Abdullah N. Kueh Y. C. Kuan G. Yahaya F. H. Lee Y. Y. (2022). A theory planned behaviour of study on improving abdominal bloating among the Malays population: a qualitative study. Malays. J. Med. Sci. 29, 77–88. doi: 10.21315/mjms2022.29.6.8, 36818903 PMC9910364

[ref2] Ajzen I. (1985). “From intentions to actions: a theory of planned behavior,” in Action control, eds. J. Kuhl and J. Beckmann (Berlin, Heidelberg: Springer), 11–39. doi: 10.1007/978-3-642-69746-3_2

[ref1101] Ajzen I. (1991). The theory of planned behavior. Organizational behavior and human decision processes 50, 179–211. doi: 10.1016/0749-5978(91)90020-T

[ref3] Ajzen I. Driver B. L. (1992). Application of the theory of planned behavior to leisure choice. J. Leisure Res. 24, 207–224. doi: 10.1080/00222216.1992.11969889

[ref4] Akçakese A. Tükel Y. Demirel M. Ertavukcu S. Deler H. (2025). The role of leisure attitude in adolescent exam anxiety and perceived stress: a gender-based analysis using the transactional model of stress and coping. Curr. Psychol. 44, 14960–14971. doi: 10.1007/s12144-025-08219-7

[ref5] Akgül B. M. Gürbüz B. (2011). Leisure attitude scale: the study of reliability and validity. Gazi J. Phys. Educ. Sport Sci. 16, 37–43. Available online at: https://izlik.org/JA54EN97YM

[ref6] Alexandris K. (2013). Segmenting recreational tennis players according to their involvement level: a psychographic profile based on constraints and motivation. Manag. Leisure 18, 179–193. doi: 10.1080/13606719.2013.796178

[ref7] Arnold E. R. Liddelow C. Vella S. A. (2024). Exploring a mother's engagement in team sport: an application of an extended theory of planned behaviour. J. Sci. Med. Sport 27, 786–792. doi: 10.1016/j.jsams.2024.06.010, 38997902

[ref8] Bandura A. (1977). Self-efficacy: toward a unifying theory of behavioral change. Psychol. Rev. 84:191. doi: 10.1016/0146-6402(78)90002-4 847061

[ref9] Bless H. Hamilton D. L. Mackie D. M. (1992). Mood effects on the organization of person information. Eur. J. Soc. Psychol. 22, 497–509. doi: 10.1002/ejsp.2420220507

[ref10] Budak D. Yumuk E. D. (2025). Leisure involvement, social inclusion, and happiness in Türkiye: a mediational analysis. Front. Public Health 13:1696910. doi: 10.3389/fpubh.2025.1696910, 41383306 PMC12689886

[ref11] Chang Y. C. Yeh T. M. Pai F. Y. Huang T. P. (2018). Sport activity for health!! The effects of karate participants’ involvement, perceived value, and leisure benefits on recommendation intention. Int. J. Environ. Res. Public Health 15:953. doi: 10.3390/ijerph15050953, 29748459 PMC5981992

[ref12] Chatzisarantis N. L. Hagger M. S. (2005). Effects of a brief intervention based on the theory of planned behavior on leisure-time physical activity participation. J. Sport Exerc. Psychol. 27, 470–487. doi: 10.1123/jsep.27.4.470

[ref13] Chein M. K. (2017). Relationship between leisure constraints, leisure motivation and leisure satisfaction: a case study of junior colleges in southern Taiwan. Int. J. Hum. Soc. Sci. Invent. 6, 29–34.

[ref14] Chen J. Yu Z. Ni R. (2025). Leisure constraints and the negotiation of structural relationships: a case study of scuba diving enthusiasts. Front. Sports Active Living 7:1586601. doi: 10.3389/fspor.2025.1586601, 40589845 PMC12206646

[ref15] Chun S. Lim J. Kang H. Ryu W. (2025). Facilitation or replacement: ICT use in leisure constraints negotiation during the digital transformation era. Sustainability 17:1503. doi: 10.3390/su17041503

[ref16] Crawford D. W. Jackson E. L. Godbey G. (1991). A hierarchical model of leisure constraints. Leis. Sci. 13, 309–320. doi: 10.1080/01490409109513147

[ref17] Dann G. M. (2016). “Motivation,” in Encyclopedia of Tourism, eds. Jafari J. Xiao H. (Switzerland: Springer International Publishing), 627–630.

[ref18] Deci E. L. Ryan R. M. (1985). Intrinsic motivation and self-determination in human behavior. New York, NY: Springer Science & Business Media. doi: 10.1007/978-1-4899-2271-7

[ref19] Deci E. L. Ryan R. M. (2000). The "what" and "why" of goal pursuits: human needs and the self-determination of behavior. Psychol. Inq. 11, 227–268. doi: 10.1207/S15327965PLI1104_01

[ref20] Di Battista R. Robazza C. Ruiz M. C. Bertollo M. Vitali F. Bortoli L. (2018). Student intention to engage in leisure-time physical activity: the interplay of task-involving climate, competence need satisfaction and psychobiosocial states in physical education. Eur. Phys. Educ. Rev. 25, 761–777. doi: 10.1177/1356336x18770665

[ref21] Eng J. J. Martin Ginis K. A. (2007). Using the theory of planned behavior to predict leisure time physical activity among people with chronic kidney disease. Rehabil. Psychol. 52, 435–442. doi: 10.1037/0090-5550.52.4.435

[ref22] Eskiler E. Yıldız Y. ve Ayhan C. (2019). The effect of leisure benefits on leisure satisfaction: extreme sports. Turk. J. Sport Exerc., 21, 16–20. doi: 10.15314/tsed.522984

[ref23] Feng H. Guo J. Hou L. (2023). The moderating role of individualism/collectivism in predicting male Chinese university students' exercise behavior using the theory of planned behavior. J. Men's Health 19, 10–21. doi: 10.22514/jomh.2023.066

[ref24] Fishbein M. Ajzen I. (2011). Predicting and changing behavior: The reasoned action approach. New York: Psychology Press. doi: 10.4324/9780203838020

[ref25] Freire T. Fonte C. (2007). Escala de atitudes face ao lazer em adolescentes e jovens adultos. Paideia 17, 79–87. doi: 10.1590/S0103-863X2007000100008

[ref26] Funk D. Alexandris K. McDonald H. (2008). Consumer behaviour in sport and events. London: Routledge. doi: 10.4324/9780080942858

[ref27] Godin G. Shephard R. (1985). A simple method to assess exercise behavior in the community. Canadian J. Appl. Sport Sci. 10, 141–146, 4053261

[ref28] Güçlü M. (2013). Gençlik döneminde boş zaman faaliyetlerinin yeri ve önemi. Gençlik Araştırmaları Dergisi 1, 158–169.

[ref29] Gürbüz B. Çimen Z. Aydın I. (2018). Leisure involvement scale: validity and reliability study of Turkish form. Spormetre Beden Eğitimi ve Spor Bilimleri Dergisi 16, 256–265. doi: 10.33689/spormetre.480235

[ref30] Han Y. Wang Y. Li P. Zhang B. (2025). Attitude, habit strength, and the relationship between intention and leisure-time physical activity behavior among college students. Res. Q. Exerc. Sport 96, 223–232. doi: 10.1080/02701367.2024.2389906, 39137291

[ref31] Hausenblas H. A. Carron A. V. Mack D. E. (1997). Application of the theories of reasoned action and planned behavior to exercise behavior: a meta-analysis. J. Sport Exerc. Psychol. 19, 36–51. doi: 10.1123/jsep.19.1.36

[ref32] Hsieh S. C. Spaulding A. Riney M. (2004). A qualitative look at leisure benefits for Taiwanese nursing students. Qual. Rep. 9, 604–629. doi: 10.46743/2160-3715/2004.1906

[ref33] Hubbard J. Mannell R. C. (2001). Testing competing models of the leisure constraint negotiation process in a corporate employee recreation setting. Leisure Sci. 23, 145–163. doi: 10.1080/014904001316896846

[ref34] Kerner M. S. Kurrant A. B. (2003). Psychosocial correlates to high school girls' leisure-time physical activity: a test of the theory of planned behavior. Percept. Mot. Skills 97, 1175–1183. doi: 10.2466/pms.2003.97.3f.1175, 15002861 PMC2386990

[ref35] Kim S. Haley E. Koo G. Y. (2009). Comparison of the paths from consumer involvement types to ad responses between corporate advertising and product advertising. J. Advert. 38, 67–80. doi: 10.2753/JOA0091-3367380305

[ref36] Kim J. Heo J. Dvorak R. Ryu J. Han A. (2018). Benefits of leisure activities for health and life satisfaction among Western migrants. Ann. Leisure Res. 21, 47–57. doi: 10.1080/11745398.2017.1379421

[ref37] Kim Y. J. Kang S. W. (2021). An analysis of the relationship between the modified theory of planned behavior and leisure rumination of Korean employees. Int. J. Environ. Res. Public Health 18:320. doi: 10.3390/ijerph18010320, 33406754 PMC7796416

[ref38] Ko Y. J. Kim Y. K. Kim M. K. Lee J. H. (2009). The role of involvement and identification on event quality perceptions and satisfaction: a case of US taekwondo open. Asia Pac. J. Mark. Logist. 22, 25–39. doi: 10.1108/13555851011013137

[ref39] Kokkinaki F. Lunt P. (1997). The relationship between involvement, attitude accessibility and attitude–behaviour consistency. Br. J. Soc. Psychol. 36, 497–509. doi: 10.1111/j.2044-8309.1997.tb01146.x

[ref40] Kuykendall L. Tay L. Ng V. (2015). Leisure engagement and subjective well-being: a meta-analysis. Psychol. Bull. 141, 364–403. doi: 10.1037/a0038508, 25602273

[ref41] Kyle G. Absher J. Norman W. Hammitt W. Jodice L. (2007). A modified involvement scale. Leisure Stud. 26, 399–427. doi: 10.1080/02614360600896668

[ref42] Kyle G. Chick G. (2004). Enduring leisure involvement: the importance of personal relationships. Leisure Stud. 23, 243–266. doi: 10.1080/0261436042000251996

[ref43] Latimer A. E. Martin Ginis K. A. (2005). The theory of planned behavior in prediction of leisure time physical activity among individuals with spinal cord injury. Rehabil. Psychol. 50:389. doi: 10.1037/0090-5550.50.4.389

[ref44] Lee K. Y. Park S. H. (2021). The effect of informal gatherings on sustainable participation in leisure sport activities: the case of South Korea. Sustainability 13:7734. doi: 10.3390/su13147734

[ref45] Lee C. Payne L. L. Berdychevsky L. (2020). The roles of leisure attitudes and self-efficacy on attitudes toward retirement among retirees: a sense of coherence theory approach. Leisure Sci. 42, 152–169. doi: 10.1080/01490400.2018.1448025

[ref46] Li Y. Zhao Y. Peng F. Lu Q. (2025). Negotiating leisure conflicts in urban square dancing: a Chinese Guanxi perspective. Leisure Stud., 1–21. doi: 10.1080/02614367.2025.2549979

[ref47] Liang Y. Li J. Wang Z. (2025). From leisure motivation to recreation specialization: a study on the psychological mechanism of sustainable participation of ice and snow sports participants. Front. Psychol. 16:1484550. doi: 10.3389/fpsyg.2025.1484550, 41356054 PMC12676907

[ref48] Lim Y. Kim M. (2026). Future time perspective and leisure motivation among Korean baby boomers: a latent profile analysis. Leis. Sci. 1–21. doi: 10.1080/01490400.2026.2623960, 37339054

[ref49] Lu J. Schuett M. A. (2014). Examining the relationship between motivation, enduring involvement and volunteer experience: the case of outdoor recreation voluntary associations. Leisure Sci. 36, 68–87. doi: 10.1080/01490400.2014.860791

[ref50] Luong T. B. Ho C. H. (2025). Leisure constraints and participant intention among Vietnamese migrant laborers in Taiwan: the role of leisure motivation and cross-cultural adaptation. J. Leisure Res. 56, 822–850. doi: 10.1080/00222216.2024.2419403

[ref51] Ma S. M. Chen S. F. Ma S. C. (2024). Relationships between social capital, leisure constraints, leisure negotiation, and running club participation. J. Leisure Res. 55, 398–419. doi: 10.1080/00222216.2023.2214559

[ref52] Morris N. J. Orton-Johnson K. (2024). Camping at home: escapism, self-care, and social bonding during the COVID-19 pandemic. Ann. Leisure Res. 27, 14–35. doi: 10.1080/11745398.2022.2082992

[ref53] Mutlu İ. (2008). Research About People Who Do Exercise and Their Attitude to Their Spare Time-A Sample of Kayseri [Dissertation]. Türkiye: Nigde University.

[ref54] Nelson T. D. Benson E. R. Jensen C. D. (2010). Negative attitudes toward physical activity: measurement and role in predicting physical activity levels among preadolescents. J. Pediatr. Psychol. 35, 89–98. doi: 10.1093/jpepsy/jsp040, 19447878

[ref55] Nicholls J. G. (1984). Achievement motivation: conceptions of ability, subjective experience, task choice, and performance. Psychol. Rev. 91, 328–346. doi: 10.1037/0033-295X.91.3.328

[ref56] Niu H. J. Hsieh K. Y. Heish F. Y. Li M. H. Yu C. C. Lin C. T. (2025). Beyond attitude: the moderating role of sport and leisure involvement in green food consumption and sustainable agriculture. J. East Eur. Manag. Stud. 30:40221. doi: 10.31083/JEEMS40221

[ref57] Pelletier L. G. Vallerand R. J. Green-Demers I. Blais M. R. Brière N. M. (1996). Vers une conceptualisation motivationnelle multidimensionnelle du loisir: Construction et validation de l'échelle de motivation vis-à-vis des loisirs (EML). Loisir et société/Society and Leisure 19, 559–585. doi: 10.1080/07053436.1996.10715532

[ref58] Ragheb M. G. Beard J. G. (1982). Measuring leisure attitude. J. Leis. Res. 14, 155–167. doi: 10.1080/00222216.1982.11969512

[ref59] Ramlan M. A. Yee N. S. Zainal Abidin Z. A. (2023). Social acceptance towards person with mobility impairment participation in nature-based recreation activities based on the theory of planned behaviour. World Leis. J. 65, 390–407. doi: 10.1080/16078055.2023.2188252

[ref60] Roberts G. C. Treasure D. C. Conroy D. E. (2007). “Understanding the dynamics of motivation in sport and physical activity: An achievement goal interpretation,” in Handbook of sport psychology. 3rd Edn. eds. G. Tenenbaum and R. C. Eklund (Hoboken, NJ: John Wiley & Sons, Inc.), 3–30. doi: 10.1002/9781118270011.ch1

[ref61] Sayili U. Gunver M. G. (2025). A novel modification approach for the one sample Kolmogorov-Smirnov test in large sample size. Scand. J. Clin. Lab. Invest. 85, 287–298. doi: 10.1080/00365513.2025.2512384, 40471823

[ref62] Sönmez A. Gürbüz B. (2022). Analysis of the relationship between leisure satisfaction and adjustment to university life among university students. J. Comput. Educ. Res. 10, 481–502. doi: 10.18009/jcer.1120672

[ref63] Tabachnick B. G. Fidell L. S. (2015) in Using Multivariate Statistics, ed. Baloglu M. (Ankara: Nobel).

[ref64] Takiguchi Y. Matsui M. Kikutani M. Ebina K. (2023). Development of leisure scores according to mental, physical, and social components and investigation of their impacts on mental health. Leis. Stud. 43, 562–577. doi: 10.1080/02614367.2023.2256027, 37339054

[ref65] Tükel Y. Akçakese A. Demirel M. Torun G. (2025). The role of leisure involvement in fostering entrepreneurship orientation among Turkish women: a resource-based view. Leis. Stud. 1–16. doi: 10.1080/02614367.2025.2495260

[ref66] Vallerand R. J. (1997). Toward a hierarchical model of intrinsic and extrinsic motivation. Adv. Exp. Soc. Psychol. 29, 271–360. doi: 10.1016/S0065-2601(08)60019-2

[ref67] Wang L. Wang L. (2015). Using theory of planned behavior to predict the physical activity of children: probing gender differences. Biomed. Res. Int. 2015:536904. doi: 10.1155/2015/536904, 26649307 PMC4663291

[ref68] Wood L. Danylchuk K. (2015). The impact of constraints and negotiation strategies on involvement in intramural sport. Manag. Sport Leis. 20, 157–173. doi: 10.1080/23750472.2015.1010279

[ref69] Xue Q. Yang J. Wang H. Zhang D. (2022). How and when leisure crafting enhances college students’ well-being: a (quantitative) weekly diary study. Psychol. Res. Behav. Manag. 15, 273–290. doi: 10.2147/prbm.s344717, 35210877 PMC8857993

[ref70] Yerlisu Lapa T. Yağar G. (2015). “Validity and reliability study of leisure time exercise questionnaire into Turkish,” in Proceedings of the 2nd International Sports Sciences Tourism and Recreation Student Congress 2015 May 28-30, ed. Ocak Y. (Afyon, Türkiye), 74–75.

[ref71] Yerlisu-Lapa T. (2014). Leisure negotiation strategies scale: a study of validity and reliability for university students. S. Afr. J. Res. Sport Phys. Educ. Recreat. 36, 201–215. Available online at: https://hdl.handle.net/10520/EJC163538

[ref72] Zhou B. Zhu Y. Huang M. Qiao G. Ryan C. Wang Y. (2025). Hiker's leisure involvement and mental health: moderating role of self-efficacy and social support. J. Outdoor Recreat. Tour. 49:100857. doi: 10.1016/j.jort.2025.100857

